# A case of urinary bladder metastasis of hepatocellular carcinoma following use of immunotherapy/tyrosine kinase inhibitor

**DOI:** 10.1002/iju5.12625

**Published:** 2023-08-13

**Authors:** Keiichiro Miyajima, Fumihiko Urabe, Tsuzuki Shunsuke, Sato Shun, Hiroyuki Takahashi, Koji Asano, Mitsuru Yanagaki, Michinori Matsumoto, Toru Ikegami, Takahiro Kimura

**Affiliations:** ^1^ Department of Urology The Jikei University School of Medicine Tokyo Japan; ^2^ Department of Pathology The Jikei University School of Medicine Tokyo Japan; ^3^ Division of Hepatobiliary and Pancreatic Surgery, Department of Surgery The Jikei University School of Medicine Tokyo Japan

**Keywords:** bladder metastasis, hepatocellular carcinoma, IO/TKI

## Abstract

**Introduction:**

Here we present a rare case of hepatocellular carcinoma metastasis to the urinary bladder in a patient with metastatic HCC.

**Case presentation:**

An 83‐year‐old man developed gross hematuria during combined treatment with an anti‐programmed death‐ligand 1 inhibitor and an anti‐vascular endothelial growth factor for metastatic HCC. A contrast‐enhanced CT revealed a 15 × 15 mm soft tissue mass protruding from the posterior bladder wall. Cystoscopy further revealed a solitary submucosal mass located on the posterior wall. The patient underwent transurethral resection of bladder tumor. The pathological findings were consistent with a diagnosis of bladder metastasis from HCC. Following a 3‐week interval after the surgical intervention, salvage therapy was resumed.

**Conclusion:**

During follow‐up after TUR‐BT in HCC patients who present with a bladder tumor, the possibility of HCC metastases to the urinary bladder should be excluded.

**Abbreviations & Acronyms**
CT: computed tomography
HCC: hepatocellular carcinoma
IO/TKI: immunotherapy/tyrosine kinase inhibitor
TUR‐BT: transurethral resection of bladder tumor

**Keynote message**

We encountered a rare case of hepatocellular carcinoma metastasis to the urinary bladder. Communication between urologists and hepatologists is essential for the appropriate sequential therapy of hepatocellular carcinoma metastasis.

## Introduction

HCC is the third most common cause of cancer‐related deaths worldwide.^1^ Most cases of HCC are diagnosed at an advanced stage as patients with early‐stage HCC typically have mild symptoms. The predominant pattern of recurrence involves liver disease whereas extrahepatic metastasis is relatively infrequent. The lungs, bones, lymph nodes, and adrenal glands are the primary sites of extrahepatic metastases.^2^ The urinary bladder is an atypical location of HCC metastasis,^3^ with only a few cases reported previously.^3^, ^4^, ^5^ Here we present a case of bladder metastasis in a patient with HCC who was treated with an IO/TKI.

## Case presentation

**Fig. 1:**
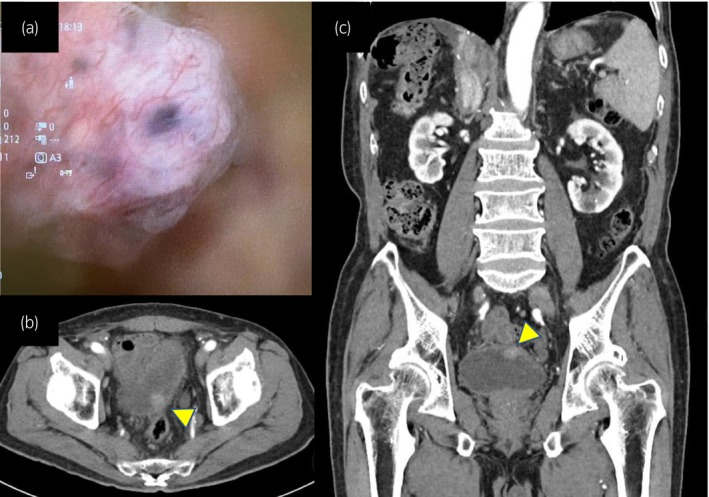
(a) Cystoscopy revealed a solitary submucosal mass on the posterior wall. (b, c) CT showing a 15 mm soft tissue mass protruding from the posterior bladder wall.

An 83‐year‐old male patient who underwent laparoscopic extended right hepatectomy for HCC 3 months previously presented with lung metastasis and suspicion of carcinomatous peritonitis in contrast‐enhanced CT. The patient was treated with atezolizumab plus bevacizumab as first‐line salvage therapy for metastatic HCC. Although the patient achieved a partial response, the lung metastasis showed progression, and bilateral hilar lymph nodes were newly detected after five cycles of the IO/TKI therapy. Thus, lenvatinib was selected as second‐line salvage therapy for metastatic HCC. During the first line of IO/TKI therapy, the patient also developed gross hematuria, and contrast‐enhanced abdominal CT revealed a 15 × 15 mm soft tissue mass protruding from the posterior wall of the bladder (Fig. 1b,c). Urine cytology yielded negative results. Cystoscopy revealed the presence of a solitary submucosal mass on the posterior wall (Fig. 1a). Based on these findings, primary bladder cancer was first suspected and surgical resection of the bladder tumor was performed.

**Fig. 2:**
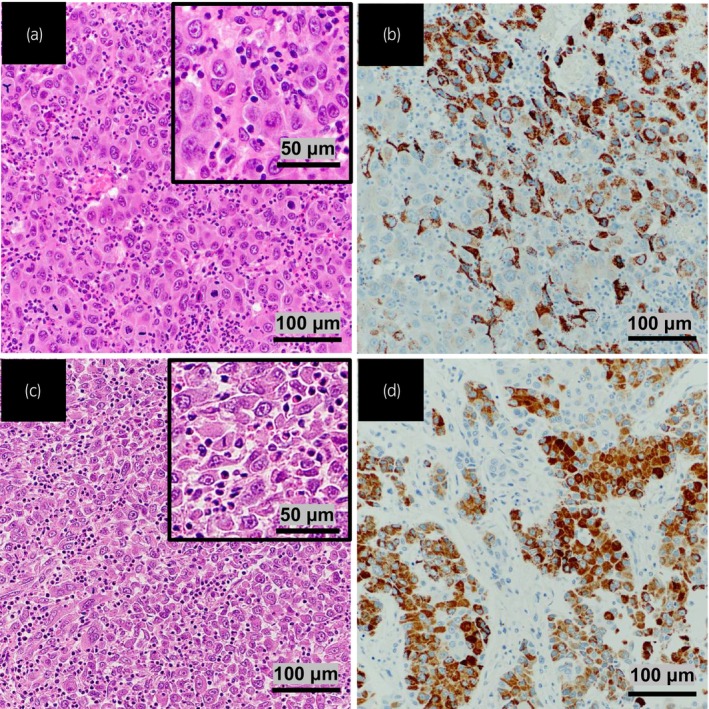
Microscopic appearance of the bladder tumor, showing a solid and sheet‐like growth of atypical epithelial cells with enlarged nuclei and prominent nucleoli (hematoxylin and eosin) (a). Immunohistochemical staining for hepatocyte paraffin 1, an HCC marker, was positive in the bladder tumor (b). These pathological findings were consistent with a diagnosis of primary hepatocellular carcinoma (c, d).

Under spinal anesthesia, the patient underwent TUR‐BT. The total surgical time was 26 min, and the patient was discharged from the hospital 4 days after TUR‐BT without experiencing any complications. Pathological examination of the TURBT specimens showed solid and sheet‐like growth of atypical epithelial cells with enlarged nuclei and prominent nucleoli in the subepithelial connective tissue and muscularis propria, similar to the histological features of HCC (Fig. 2a). Immunohistochemical staining for GATA‐binding protein 3, which is typically positive in urothelial carcinoma, was negative, whereas staining for hepatocyte paraffin 1, an HCC marker, was positive (Fig. 2b). These pathological findings were consistent with a diagnosis of primary HCC with bladder metastasis (Fig. 2c,d). Surgical margin was negative. Four weeks after the surgery, lenvatinib was resumed. During the 2‐month follow‐up after the surgery, no clinical progression of lung metastasis and bilateral hilar lymph nodes was observed.

## Discussion

The most common sites of extrahepatic metastasis in HCC are lungs, bones, lymph nodes, and adrenal glands.^2^ However, in our case, bladder metastases are observed, which are relatively rare in HCC.

Only a few cases of HCC with bladder metastases have previously been reported.^3^, ^4^, ^5^ Kim *et al*.^3^ reported a case of HCC with urinary bladder metastasis. The patient underwent transcatheter arterial chemoembolization for HCC as primary therapy. Subsequent contrast‐enhanced CT revealed the presence of a new bladder tumor during follow‐up. As a result, the patient underwent TUR‐BT, and the pathological analysis confirmed the diagnosis of metastatic HCC. Similarly, Yasutomi *et al*.^4^ reported a case of solitary metastasis of HCC to the urinary bladder following transcatheter arterial chemoembolization for primary HCC. By contrast, our patient underwent right extended hepatectomy, followed by the development of lung metastasis and carcinomatous peritonitis 3 months later. During salvage treatment using the IO/TKI combination therapy, the development of a bladder tumor and progression of lung metastasis were observed. As a result, the patient underwent TUR‐BT, and the pathological analysis revealed metastatic HCC. To the best of our knowledge, this is the first case report of bladder metastasis in unresectable HCC following the use of IO/TKI therapy.

Compared to the two previous cases,^3^, ^4^ our patient was diagnosed with a bladder tumor with advanced‐stage HCC. Chung *et al*.^5^ also reported a case of advanced HCC with bladder metastasis. In that case, the patient developed gross hematuria, and contrast‐enhanced CT revealed a bladder tumor and recurrent HCC in the liver. As a result, the patient underwent TUR‐BT, and the pathological diagnosis was consistent with metastatic HCC. However, the patient declined further treatment and died 5 months after TUR‐BT due to HCC progression.^5^ These cases suggest that bladder metastasis of HCC can occur as a solitary metastasis or multiple metastases. Primary bladder cancer and HCC metastases to the urinary bladder have similar morphology in imaging and cystoscopy.^3^, ^5^ Therefore, histological evaluation is necessary to distinguish between these entities. Furthermore, the microenvironment at the metastatic site facilitates the development of organ‐specific metastatic cells with a high capacity for aggressive colonization. The bladder poses unique selective pressures for the development of metastasis, which are distinct from those for conventional sites, such as lungs, bones, and adrenal glands. Therefore, further investigations are needed to understand the mechanisms underlying bladder metastases. Appropriate exchange of information and effective communication between urologists and hepatologists is essential for the selection of the appropriate sequential therapy following TUR‐BT.

In conclusion, we present a rare case of HCC metastasis to the urinary bladder. Although bladder metastases are relatively rare, their detection is essential for the appropriate treatment of patients with HCC metastasis in the urinary bladder by urologists and hepatologists.

## Author contributions

Keiichiro Miyajima: Conceptualization; data curation; writing – original draft. Fumihiko Urabe: Conceptualization; supervision; visualization; writing – original draft. Tsuzuki Shunsuke: Supervision; writing – review and editing. Sato Shun: Supervision; visualization; writing – review and editing. Hiroyuki Takahashi: Supervision; visualization; writing – review and editing. Koji Asano: Supervision; writing – review and editing. Mitsuru Yanagaki: Supervision; visualization; writing – review and editing. Michinori Matsumoto: Supervision; writing – review and editing. Toru Ikegami: Validation; visualization; writing – review and editing. Takahiro Kimura: Supervision; writing – review and editing.

## Conflict of interest

The authors declare that they have no conflicts of interest to report.

## Approval of the research protocol by an Institutional Reviewer Board

Not applicable.

## Informed consent

Consent to participate and for publication were acquired from the patient.

## Registry and the Registration No. of the study/trial

Not applicable.
